# Adjunctive Thymosin Beta-4 Treatment Influences PMN Effector Cell Function during *Pseudomonas aeruginosa*-Induced Corneal Infection

**DOI:** 10.3390/cells10123579

**Published:** 2021-12-18

**Authors:** Yuxin Wang, Thomas W. Carion, Abdul Shukkur Ebrahim, Gabriel Sosne, Elizabeth A. Berger

**Affiliations:** Department of Ophthalmology, Visual and Anatomical Sciences, School of Medicine, Wayne State University, Detroit, MI 48201, USA; gp8667@wayne.edu (Y.W.); tcarion@med.wayne.edu (T.W.C.); eabdulsh@med.wayne.edu (A.S.E.); gsosne@med.wayne.edu (G.S.)

**Keywords:** Tβ4, neutrophils/PMN, immunoregulation, keratitis, ROS, NETosis, apoptosis

## Abstract

Previous work examining the therapeutic efficacy of adjunct thymosin beta 4 (Tβ4) to ciprofloxacin for ocular infectious disease has revealed markedly reduced inflammation (inflammatory mediators and innate immune cells) with increased activation of wound healing pathways. Understanding the therapeutic mechanisms of action have further revealed a synergistic effect with ciprofloxacin to enhance bacterial killing along with a regulatory influence over macrophage effector cell function. As a natural extension of the aforementioned work, the current study uses an experimental model of *P. aeruginosa*-induced keratitis to examine the influence of Tβ4 regarding polymorphonuclear leukocyte (PMN/neutrophil) cellular function, contributing to improved disease response. Flow cytometry was utilized to phenotypically profile infiltrating PMNs after infection. The generation of reactive oxygen species (ROS), neutrophil extracellular traps (NETs), and PMN apoptosis were investigated to assess the functional activities of PMNs in response to Tβ4 therapy. In vitro work using peritoneal-derived PMNs was similarly carried out to verify and extend our in vivo findings. The results indicate that the numbers of infiltrated PMNs into infected corneas were significantly reduced with adjunctive Tβ4 treatment. This was paired with the downregulated expression of proinflammatory markers on these cells, as well. Data generated from PMN functional studies suggested that the corneas of adjunctive Tβ4 treated B6 mice exhibit a well-regulated production of ROS, NETs, and limited PMN apoptosis. In addition to confirming the in vivo results, the in vitro findings also demonstrated that neutrophil elastase (NE) was unnecessary for NETosis. Collectively, these data provide additional evidence that adjunctive Tβ4 + ciprofloxacin treatment is a promising option for bacterial keratitis that addresses both the infectious pathogen and cellular-mediated immune response, as revealed by the current study.

## 1. Introduction

Microbial keratitis is a sight-threatening infection of the cornea often resulting from ocular injury or extended contact lens wear [1,2]. *Pseudomonas (P.) aeruginosa* is one of the most frequently isolated pathogens to induce microbial keratitis and is a major cause of corneal blindness worldwide [2,3]. *P. aeruginosa*-induced corneal infections are more destructive than any other bacterial pathogen, causing severe complications including corneal ulcers, corneal perforation, scarring, and eventual blindness [4,5]. The pathogenesis related to microbial keratitis involves both the host and bacteria [6]. Regarding the latter, *P. aeruginosa* can produce various cell-associated and extracellular virulence factors including toxins and proteases to trigger and sustain the infection, leading to tissue damage [7]. Another principal factor contributing to the destruction of the cornea during microbial keratitis is the over-activation of the host defense system [8]. *P. aeruginosa* can stimulate corneal epithelial cells to produce inflammatory mediators by activating different immune system pathways during infection. Chemical mediators, such as cytokines and chemokines, recruit leukocytes, mainly PMNs, to the site of infection to phagocytose and effectively remove the pathogen [9]. PMNs are the most abundant cellular effectors in the acute phase of inflammation, although macrophages also play a critical function [10]. Besides releasing inflammatory mediators, PMNs form corneal phagolysosomes to engulf the pathogen and degranulate lysosomal enzymes [11]. In addition, PMNs undergo oxidative burst to generate ROS, including superoxide anions, that are directly toxic to bacteria [10]. In addition, excessive levels of ROS can induce lipid peroxidation upon contact with membrane phospholipids resulting in the formation of toxic breakdown products that alter cellular function and cause tissue degradation [12]. Another more recently recognized powerful effector mechanism of PMNs is the release of neutrophil extracellular traps or NETs to capture and kill pathogens. There are at least three diverse mechanisms by which NETs are formed: the classical or suicidal NETosis, the noncanonical pathway, and the vital NETosis. NETosis can by triggered by toll-like receptors (TLRs), complement receptors, lipopolysacchride (LPS) of Gram-negative bacteria, and other chemical stimuli [13]. Depending on the trigger, this process can be mediated by NADPH oxidase and the subsequent intracellular release of myeloperoxidase (MPO) and neutrophil elastase (NE) [14]. The nuclear translocation of these enzymes allows for cleavage and citrullination of histone and stepwise chromatin decondensation. Chromatin is then expelled into the extracellular space by gasdermin D (GSDMD)-driven membrane pores to trap pathogens [14,15,16,17]. Ideally, when the cellular functions of PMNs are fulfilled, spontaneous apoptosis will occur to preserve PMN membrane integrity and prevent the release of intracellular toxic components that would otherwise damage surrounding tissues. The subsequent clearance of apoptotic PMNs is critical to the successful resolution of inflammation. Alternatively, secondary necrosis and chronic inflammation will occur [18]. To this extent, recruited PMNs are beneficial for the host to eradicate the invaded pathogens. However, the problem related to ocular infections is that active PMNs are chronically and overwhelmingly recruited. This exacerbated presence of PMNs and excessive release of PMN-derived end products are extremely toxic to the cornea, contributing to tissue destruction and subsequent loss of vision [9,10,19]. 

Therapeutic options for bacterial keratitis remain limited. Topical antibiotics remain the only evidence-based treatments indicated to preserve visual acuity [19,20]. However, due to increasing multidrug resistance, there is concern regarding antibiotic efficacy against corneal pathogens [21,22]. Further, the use of adjunctive corticosteroids remains unsubstantiated by studies that indicate no statistical improvement in the clinical outcomes of *P. aeruginosa*-induced keratitis compared to the placebo group [3]. Proponents for the administration of topical corticosteroids in bacterial keratitis declare that this class of steroid hormones can suppress inflammation and reduce subsequent corneal scarring and vision loss. However, the potential disadvantages are delayed epithelial healing, exacerbated infection, local immunosuppression, and predisposed corneal melting [3,4]. In clinical medicine, identifying new therapies that effectively remove the infectious pathogen, while immunoregulating the host response is urgently needed to improve patient outcomes and public health.

Tβ4 is a naturally occurring, small peptide with 43 amino acids. It is present in all cell types except red blood cells and detected within all body fluid with extremely high concentrations in wound fluid and platelets [23,24]. Tβ4 has been appreciated for multiple functions, such as modulating inflammatory responses, enhancing the migration of endothelial and epithelial cells, and suppressing apoptosis and oxidative damage [25,26,27,28,29]. Tβ4 also promotes wound healing by regulating fibronectin:integrin and uPA:uPAR [30] and assembling the distribution of collagen in wounded tissues [31]. Anti-inflammatory and immunoregulatory functions of Tβ4 have been shown to markedly improve the outcomes of ocular surface diseases [30,32]. An investigation into Tβ4′s influence during microbial keratitis has most recently revealed an effect on macrophage cellular function [33]. This study continues our work regarding the mechanism(s) of Tβ4 by examining the impact on PMN function using a combination of in vivo and in vitro approaches. 

## 2. Materials and Methods

### 2.1. Bacterial Preparation

Bacterial cultures were prepared as previously described [34]. Briefly, *P. aeruginosa* cytotoxic strain 19660 was grown in peptone-tryptic soy broth (PTSB) containing 5% peptone (BD Biosciences, Franklin Lakes, NJ, USA) at 37 °C in a rotary shaking water bath (Polysciences, Niles, IL, USA) at 150 rpm for ~18 h to an optical density (OD) of 1.4 to 1.6 at 540 nm (~10^8^ CFU/µL) as determined by spectrophotometer (ThermoFisher Scientific, Rockford, IL, USA). Stock cultures were then centrifuged at 6000× *g* for 10 min at 4 °C, washed once with sterile normal saline, and resuspended in saline to a concentration of 10^6^ CFU/µL for use as indicated below.

### 2.2. Experimental Animal Protocol

Eight-week-old female C57BL/6 (B6) mice (Jackson Laboratory, Bar Harbor, ME, USA) were used in the current study. The central cornea of the left eye was scarified as previously described [34]. An aliquot of bacterial suspension (5 μL containing 5 × 10^6^ CFU of *P. aeruginosa* 19660) was administered topically to the surface of the scarified cornea. Mice were randomized into four different treatment groups consisting of: PBS (controls), Tβ4 alone (0.1%; Regenerx Biopharmaceuticals Inc., Rockville, MD, USA), ciprofloxacin alone (0.3%—the current standard concentration used clinically; Akron, Inc. Lake Forest, IL, USA), or Tβ4 + ciprofloxacin. Treatments were topically applied to the cornea (5 μL) 3× per day and initiated 24 h p.i. Additionally, uninfected corneas of naïve mice were used as a control where appropriate, as indicated. All animals were treated as authorized by Wayne State University Institutional Animal Care and Use Committee (protocol 19-10-1312). Treatment of animals used in this study conformed to the Association for Research in Vision and Ophthalmology’s statement on the Use of Animals in Ophthalmic and Vision Research.

### 2.3. PMN Isolation and Treatment

Peritoneal-derived PMNs were collected from B6 mice as previously described [35,36,37,38]. In brief, mice received an i.p. injection of a 9% casein solution (1.0 mL), followed by a second injection 24 h later. Peritoneal lavage was carried out three hours after the second injection. PMNs were collected with harvest solution (0.02% EDTA in 1× PBS) (Sigma-Aldrich, St. Louis, MO, USA), washed, and isolated from peritoneal exudate cells with a 90% Percoll gradient. Viable cells (>95%) were counted and resuspended in RPMI 1640 with 10% FBS (ThermoFisher Scientific, Rockford, IL, USA). PMNs were seeded in six-well plates at a concentration of 1 × 10^6^ cells/well. Cells were subsequently stimulated with lipopolysaccharide (25 µg/mL) (PA, serotype 10-derived LPS, Sigma-Aldrich, St. Louis, MO, USA) and then immediately treated with Tβ4 (final concentration is 0.1%), ciprofloxacin (final concentration is 0.03%), and adjunct Tβ4 (final concentration of Tβ4 and ciprofloxacin at 0.1% and 0.03%, respectively) for 24 h. Cell supernatants and lysates were collected for assays.

### 2.4. Flow Cytometric Analyses

Individual corneas from B6 mice (*n* = 5/group) were excised at 3 days p.i., incubated in 250 µL RPMI 1640 containing DNase I and collagenase at 37 °C for 1 h, then manually homogenized to obtain single-cell suspensions [39]. Cells were washed using 1% BSA FACS buffer. Trypan blue was used to assist cell counting and viability. Cell suspensions were then incubated with antibodies indicated below along with a fixable viability cell stain and staining buffer at 4 °C for 30 min. Cells were washed 2× and resuspended in 1 mL cold FACS buffer. Samples were immediately acquired using a flow cytometer (LSRFortessa; Beckton Dickinson, San Jose, CA, USA). Analysis of the data was carried out using FlowJo software (Ashland, OR, USA). The following conjugated antibodies were used for cell surface staining: CD45 (30-F11) (BD Biosciences, San Jose, CA, USA), Ly6G (1A8) and F4/80 (BM8) (BioLegend, San Diego, CA, USA) were utilized as gating markers; CD13 (R3-63) (Novus Biologicals, Centennial, CO, USA), CD63 (NVG-2), CD177 (Y127) (BD Biosciences, San Jose, CA, USA), CD80 (16-10A1) (BioLegend, San Diego, CA, USA), AnxA1 (7H46L26), CD206 (MR6F3), and CD192 (SA203G11) (BioLegend, San Diego, CA, USA) to determine activation state; and LIVE/DEAD™ Fixable Aqua dead cell stain (L34965) (ThermoFisher Scientific, Rockford, IL, USA) was used as an exclusion dye. All antibodies were used at the dilution range recommended by the manufacturer.

### 2.5. Measurement of ROS and Superoxide

Individual corneas were homogenized in RIPA buffer (Cell Signaling Technology, Danvers, MA, USA). PMNs collected from in vitro assays were lysed in RIPA buffer, as well. A protease inhibitor cocktail (Thermo Fisher Scientific, Waltham, MA, USA) was added to all samples. Individual corneal lysates (3 days p.i.) and PMN cell lysates (24 h after stimulation) from each experimental group were centrifuged at 12,000 rpm for 20 min, then supernatants were collected and normalized for equal amounts of protein as determined by BCA methods. Corneal lysates (20 µg) and PMN cell lysates (10 µg) from each group were incubated in reaction buffer (130 mM KCl, 5 mM MgCl_2_, 20 mM NaH_2_PO_4_, 20 mM Tris-HCL, pH 7.4, 30 mM D-glucose, 7.5 μM 2′,7′-dichlorofluorescein diacetate [DCFH-DA; ThermoFisher Scientific, Waltham, MA, USA], and dihydroethidium [DHE; Sigma-Aldrich, St. Louis, MO, USA]) for 1 h at 37 °C, as previously carried out [33,40,41]. Reaction buffer excluding DCFH-DA or DHE dyes were used as negative controls. The SpectraMax M3 Multi-Mode reader (Molecular Devices, Sunnyvale, CA, USA) was utilized to measure detectable DCF and ethidium as the fluorescent products of oxidized DCFH-DA and DHE, respectively. Wavelengths for excitation and emission were 485 and 527 nm for DCFH-DA and 520 and 610 nm for DHE [42]. Final fluorescent values were calculated by subtracting negative control values from DCF or ethidium fluorescent values with results reported as fluorescence intensity ± SD. 

### 2.6. Protein Analysis

Individual corneal and PMN cell lysates were prepared for lipid peroxidation [hexanoyl-lysine adduct (HEL)], NETosis, and apoptosis analysis. BCA method (Thermo Fisher Scientific, Waltham, MA, USA) was used to determine total protein concentrations. Protein was loaded in equal amounts (15 µg/well) and separated using 4% to 20% tris-glycine gels (Invitrogen, Waltham, MA, USA). After transferring to PVDF membranes, 5% nonfat milk dissolved in TBST (10 mmol/L Tris-HCl buffer, pH 8.0, 150 mmol/L NaCl, and 0.1% Tween 20) was used for blocking by incubating at room temperature for 60 min. Incubation of membranes was carried out overnight at 4 °C with antigen-specific 1° antibodies and included: antiHEL (1:500; AdipoGen Life Sciences, San Diego, CA, USA), antiNE (1:500; Abcam, Cambridge, UK), anticaspase 3, anticaspase 8, antiBax, and antiBcl-2 (1:1000; Cell Signaling Technology, Danvers, MA, USA); and anti–β-actin (1:1000; Santa Cruz Biotechnology, Dallas, TX, USA). After washing 5× with TBST, blots were incubated with species-specific horseradish peroxidase-conjugated secondary antibodies for 1 h at room temperature. Images were visualized by incubation with a chemiluminescence substrate kit (Thermo Fisher Scientific, Waltham, MA, USA) and collected (Bio-Rad Molecular Imager, ChemiDoc XRS+, Hercules, CA, USA). β-actin was used as the loading control. Quantification of protein expression was based on the densitometry of blots by utilizing the Image Studio Lite software version 5.2 (LI-COR Biosciences, Lincoln, NE, USA) after normalizing to β-actin. One representative blot is displayed for each protein.

### 2.7. Enzyme-Linked Immunosorbent Assays

Normal (uninfected) and infected corneas, and PMN cell lysates were collected from each treatment group. Samples were homogenized in RIPA buffer (Cell Signaling Technology, Danvers, MA, USA) together with a protease inhibitor cocktail (Thermo Fisher Scientific, Waltham, MA, USA). Digested samples were centrifuged at 5000× *g* for 5 min, and an aliquot of each supernatant was assayed in triplicate for cell death detection (Sigma-Aldrich, St. Louis, MO, USA) and citrullinated histone H3 (Cayman Chemical, Ann Arbor, MI, USA) per the manufacturer’s instructions. The absorbance of cell death detection was measured at 405 nm, and the enrichment factor (EF) was calculated based on the provided formula. The reported sensitivity of citrullinated histone H3 is 0.3 ng/mL and data are presented as average ng/mL ± SD.

### 2.8. Statistical Analysis

A minimum of three independent experiments was used for each assessment. The data shown are representative of results obtained and are presented as mean ± SD unless indicated otherwise. Results were obtained in a blinded fashion to increase rigor. All data were analyzed using one-way ANOVA, then Bonferroni’s multiple comparison test (GraphPad Prism, San Diego, CA, USA) as a post hoc method, with the significance defined as *p* < 0.05.

## 3. Results

### 3.1. Flow Cytometric Analyses of PMN Infiltrate in P. aeruginosa Infected B6 Mice

**Figure 1:**
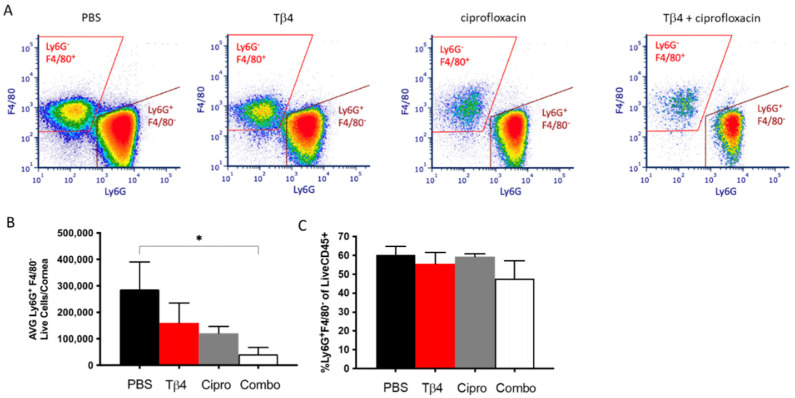
Flow cytometry results of PMN cellular infiltrates following *P. aeruginosa*-induced corneal infection. CD45^+^ Ly6G^+^ F4/80^−^ leukocytes identified as PMNs as determined by flow cytometry at 3 days p.i. in corneas of PBS-, Tβ4-, cipro-, and adjunct Tβ4-treated B6 mice (**A**). Results are shown as averages of single cell populations (**B**) and % of live PMNs per total population of live leukocytes (**C**). *n* = 5 corneas/group; * *p* < 0.05.

We have previously shown that adjunctive Tβ4 treatment reduces PMN infiltration into infected corneas of B6 mice as indirectly indicated by MPO assay [30]. In the current study, flow cytometry was explicitly used to investigate PMN infiltration into corneas of each treatment group at three days p.i. as shown in Figure 1. We have previously published that significantly fewer CD45^+^ leukocytic cells were detected in the adjunctive Tβ4 group when compared to PBS controls with a significant reduction in % of CD45^+^ cells per total cells of the cornea [33]. Live PMN (CD45^+^/Ly6G^+^/F480^−^) singlet populations were further characterized from the CD45^+^ gated population (Figure 1A). Gating strategies are presented as Supplementary Material—Figure S1. Despite trending downward, only the adjunctive Tβ4 treatment group showed significantly reduced infiltration of PMNs when compared to the PBS control (Figure 1B). There were no statistical differences when calculated as the % of PMN/total live CD45^+^ cells indicating that PMNs comprised the same % of CD45^+^ cells across all groups (Figure 1C). 

### 3.2. Adjunctive Tβ4 Treatment Influences Phenotypic Profiles of PMN in the Infected Cornea

**Figure 2:**
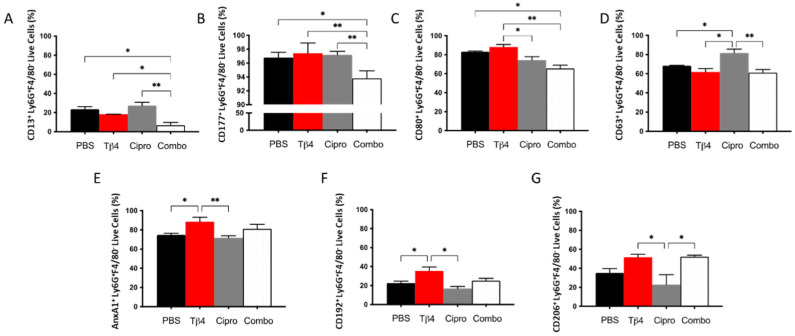
Expression of cell surface markers as detected on PMNs by flow cytometry. Select proinflammatory molecules CD13 (**A**), CD177 (**B**), CD80 (**C**), and CD63 (**D**) and anti-inflammatory molecules AnxA1 (**E**), CD192 (**F**), and CD206 (**G**) were detected on CD45^+^ Ly6G^+^ F4/80^−^ PMNs from 3 day-infected corneas of PBS-, Tβ4-, cipro-, and adjunct-treated B6 mice. Results are shown as averages of singlet populations for CD45^+^ Ly6G^+^ F4/80^−^ PMNs. *n* = 5 corneas/group; * *p* < 0.05, ** *p* < 0.01.

To begin exploring the underlying influence of Tβ4 on the activation state of neutrophils, proinflammatory (CD13, CD177, CD80, and CD63) and anti-inflammatory (annexin A1 [AnxA1], CD192, and CD206) cell surface markers were further assessed by flow cytometry in the PMN subpopulation following infection. As presented in Figure 2, the % of PMN expressing proinflammatory molecules CD13 (A) and CD177 (B) were both significantly downregulated in the adjunctive Tβ4 treated corneas when compared to all other treatment groups. CD80 (C) was significantly reduced in the adjunctive Tβ4 treatment group compared to the PBS and Tβ4 only groups, but not ciprofloxacin. While ciprofloxacin alone upregulated the expression of CD63 (D) when compared to the PBS control and both Tβ4 only and adjunctive Tβ4 treatments. Regarding anti-inflammatory cell surface markers, % of PMN expressing AnxA1(E) and CD192 (F) were significantly enhanced in the Tβ4 only treatment groups. An increasing trend of AnxA1 and CD192 was observed in the adjunctive Tβ4 corneas albeit without significant differences when compared to other groups. Surprisingly, ciprofloxacin significantly reduced AnxA1 and CD192 when compared to Tβ4 only, and CD206 (G) against both Tβ4 and adjunct Tβ4 treatments. These flow cytometry results together suggest that Tβ4 treatment of *P. aeruginosa*-infected corneas affects the activation state of PMNs by decreasing the expression of pro-inflammatory molecules and enhancing anti-inflammatory markers. 

### 3.3. Adjunctive Tβ4 Treatment Inhibits ROS Generation and Lipid Peroxidation

**Figure 3:**
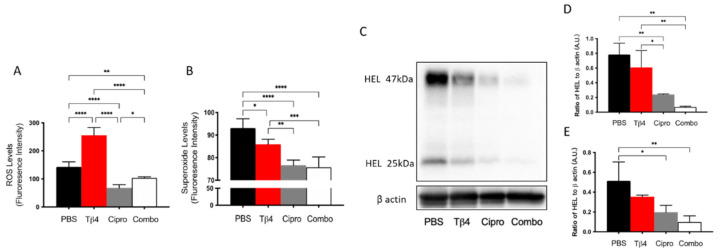
In vivo assessment of oxidative stress in corneal lysates following 3 days of *P. aeruginosa*-induced infection. Results for ROS (**A**) and superoxide production (**B**) are presented as mean fluorescence intensity ± SD. Western blot was used to detect the reactive species-induced lipid peroxidation damage as determined by HEL adduct formation (**C**). Densitometric analysis of HEL adducts formed at approximately 47 kDa (**D**) and 25 kDa (**E**). Data shown are representative of three independent experiments and normalized to β-actin ± SD. *n* = 3 corneas/group; * *p* < 0.05; ** *p* < 0.01; *** *p* < 0.001; and **** *p* < 0.0001.

One of the principal mechanisms of PMNs to fight against pathogens is to release ROS [43]. Conversely, large amounts of ROS generated in dysregulated inflammation is the primary pathogenic mechanism inducing inflammatory damage [44]. Therefore, potential differences in ROS levels (A) between treatment groups were examined at three days p.i. as shown in Figure 3. Tβ4 only resulted in a tremendous increase in the production of ROS when compared to all other groups. Both the ciprofloxacin and adjunctive Tβ4 treatment groups significantly hindered ROS generation compared to the PBS control. 2′,7′-dichlorofluorescin diacetate (DCFH-DA) is oxidized by ROS, resulting in the stable fluorescent end product, dichlorofluorescein (DCF) [45]. The findings revealed that all treatment groups significantly inhibited superoxide production compared to the PBS control, as indicated by DCF measurement (B). Dihydroethidium (DHE), a freely permeable ROS-specific fluorescent dye, is oxidized to ethidium by superoxide radicals (O_2_^−^) [45]. Following incubation with DHE, superoxide was measured in infected corneas, as well. Remarkably, both ciprofloxacin and the adjunctive Tβ4 treatment groups further decreased superoxide levels when compared to Tβ4 alone. These findings were then confirmed by detecting HEL adduct formation (C–E), which takes place during the early stages of ROS-induced lipid peroxidation [46]. PBS controls revealed strong lipid peroxidation with detectable HEL adduct formation at approximately 25 kDa (D) and 47 kDa (E); no differences were detected after Tβ4 alone treatment. Whereas both ciprofloxacin and adjunctive Tβ4 treatments resulted in significantly blunted HEL adduct formation, as quantified by a densitometric analysis of the two predominate bands. These results further illustrate that adjunctive Tβ4 treatment not only influences the phenotypical profiles of PMNs but also inhibits a key cellular function in the corneas of *P. aeruginosa*-infected mice. 

### 3.4. Adjunctive Tβ4 Treatment Inhibits In Vitro ROS Generation and Lipid Peroxidation by PMN 

**Figure 4:**
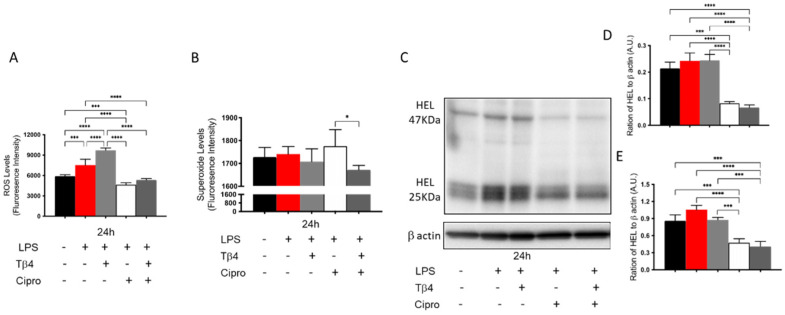
In vitro assessment of ROS, superoxide production, and lipid peroxidation in peritoneal-isolated PMN following 24 h of LPS stimulation. For ROS (**A**) and superoxide (**B**) assessments, results are presented as mean fluorescence intensity ± SD of three independent experiments in triplicate. Lipid peroxidation damage as determined by HEL adduct formation (**C**) was assessed by Western blot. Densitometric analysis of HEL adducts formed at approximately 47 kDa (**D**) and 25 kDa (**E**). Data shown are representative of three independent experiments and normalized to β-actin ± SD. *n* = 3; * *p* < 0.05; *** *p* < 0.001; and **** *p* < 0.0001.

Many different types of inflammatory cells function together in response to corneal infections. Carrying out in vitro examination of PMNs allows for the exclusion of confounding factors, particularly other inflammatory cells and the presence of bacteria. Therefore, complimentary in vitro studies were conducted to further confirm the in vivo findings regarding how Tβ4 affects PMN cellular function (Figure 4A–E) using LPS, a major component of the Pseudomonas cell wall known to be a robust virulence factor and activator of TLR4, which plays a key role in neutrophil activation [47]. LPS-induced ROS and HEL adduct levels were evaluated at 24 h after stimulation by using peritoneal-derived PMNs from B6 mice. The results reveal that the production of ROS (A) significantly increased after LPS stimulation and that Tβ4 only treatment, similar to in vivo findings, enhanced ROS generation when compared to all groups at 24 h. However, the ciprofloxacin and adjunctive Tβ4 treatment groups significantly inhibited LPS-induced ROS levels. Superoxide levels (B) were similarly expressed among all experimental groups except for a significant decrease observed between the adjunctive Tβ4 and ciprofloxacin only groups. The inhibitory effect of adjunctive Tβ4 was further revealed through significantly decreased HEL adduct formation (C–E). The HEL adducts, formed at approximately 47 kDa and 25 kDa, were uniformly expressed among media only, LPS only, and Tβ4 only groups. Notably, both the ciprofloxacin and adjunctive Tβ4 treatments significantly inhibited LPS-induced HEL adduct formation at 24 h. These data demonstrate that both ciprofloxacin and adjunctive Tβ4 treatments effectively inhibit the generation of ROS after LPS stimulation, which protects activated PMNs from subsequent ROS-induced membrane damage; however, it is likely that this particular effect may be due to ciprofloxacin alone as demonstrated by both the in vivo and in vitro assessments.

### 3.5. Adjunctive Tβ4 Treatment Downregulates NETosis Leading to Improved Disease Response

**Figure 5:**
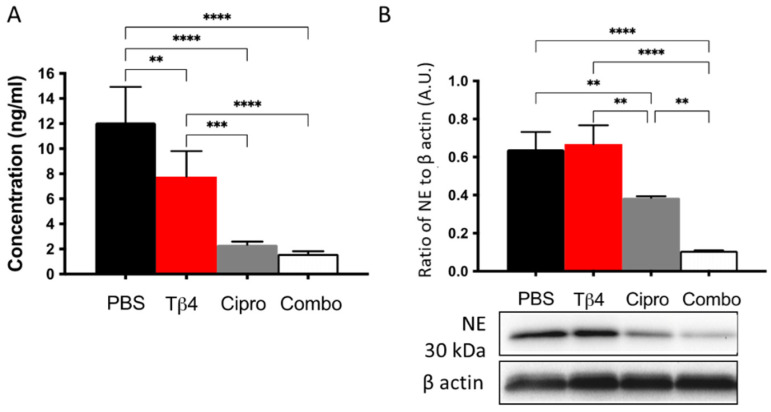
In vivo assessment of NETosis activity following *P. aeruginosa*-induced corneal infection. Citrullinated histone H3 concentration was detected from corneal lysates at 3 d p.i. by the ELISA (**A**). Data are reported as mean concentration (ng/mL) ± SD of three independent experiments in the PBS control, Tβ4-, cipro-, and adjunct-Tβ4 groups. Protein levels of NE were measured by Western blot (**B**). Results shown are representative of three independent experiments and normalized to β-actin ± SD. *n* = 3; ** *p* < 0.01; *** *p* < 0.001; and ****p < 0.0001.

PMNs can respond to pathogens by releasing NETs, which effectively trap and kill invading pathogens [14]. The protein levels of citrullinated histones H3 and NE, two critical components of NETosis [14], were measured to continue investigating Tβ4-induced influences on PMN cellular function. As shown in Figure 5, the results indicate that citrullinated histone H3 (A), which helps to decondense the chromosomes during NETosis, was increasingly downregulated in the corneas of Tβ4-, ciprofloxacin-, and adjunctive Tβ4-treated B6 mice following infection. Moreover, citrullinated histone H3 was further decreased in ciprofloxacin only and adjunctive Tβ4 treatment groups when compared to Tβ4 alone. During this process, NE localizes within the PMN nucleus where it cleaves histones and other proteins causing DNA decondensation—key steps to preparing cellular DNA for subsequent NET release [14]. The levels of NE (B) were similar between the PBS control and Tβ4 only treatment groups. Ciprofloxacin treatment significantly inhibited NE levels, which were further reduced after the adjunctive Tβ4 treatment when compared to all other treatment groups. These findings reveal that adjunctive Tβ4 regulates NETosis to improve disease outcome following *P. aeruginosa*-induced corneal infection.

### 3.6. Adjunctive Tβ4 Treatment Regulates NETosis In Vitro

**Figure 6:**
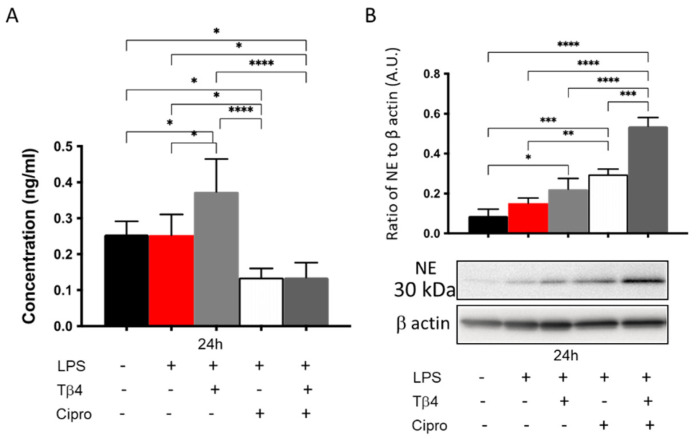
In vitro assessment of NETosis activity in peritoneal-isolated PMN following LPS stimulation. Data are shown as mean concentration (ng/mL) of H3 ± SD (**A**) or NE normalized to β-actin ± SD (**B**). Data are representative of three independent experiments. *n* = 3; * *p* < 0.05; ** *p* < 0.01; *** *p* < 0.001; and **** *p* < 0.0001.

NETosis was assessed in vitro as well to further confirm the modulatory role of adjunctive Tβ4 treatment on PMN effector cellular function after LPS stimulation. Results shown in Figure 6 reveal that LPS stimulation had no impact on citrullinated histone H3 levels (A). However, there was a significant upregulation in the Tβ4 only treatment group, while levels were remarkably inhibited by the ciprofloxacin and the adjunctive treatment groups when compared to media only, LPS only, and Tβ4 only. Protein levels of NE were analyzed by Western blot as well (B). Contrary to the in vivo results, the expression of NE in PMNs was increasingly elevated in the Tβ4 only, ciprofloxacin only, and adjunctive Tβ4 treatment groups, respectively. Tβ4 only and ciprofloxacin only significantly increased NE levels compared to media only and LPS only groups. Further, levels were significantly increased in the adjunctive Tβ4 treated peritoneal-derived PMN when compared to all other groups. These findings suggest that both ciprofloxacin and adjunctive Tβ4 treatment exert a suppressive effect regarding NETosis following LPS stimulation, but revealed differences between the in vivo and in vitro conditions, as indicated by NE levels. 

### 3.7. Adjunctive Tβ4 Treatment Enhances Proresolving Response by Regulating Apoptosis

**Figure 7:**
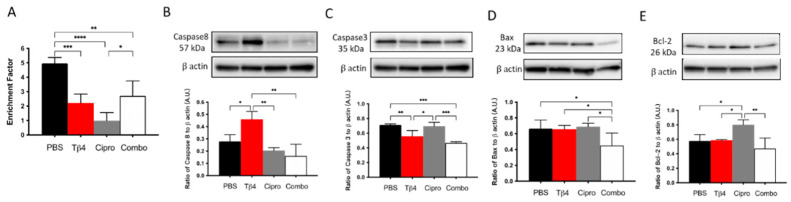
Apoptotic activity was determined using select markers as detected at the protein level. Cytoplasmic DNA fragments (**A**), caspase-8 (**B**), caspase-3 (**C**), Bax (**D**), and and Bcl-2 (**E**) were assessed in corneas of PBS-, Tβ4-, cipro-, and adjunct-treated B6 mice at 3 days after infection. Western blot results are presented as a ratio to β-actin ± SD. *n* = 3 corneas/group; * *p* < 0.05, ** *p* < 0.01; *** *p* < 0.001, and **** *p* < 0.0001.

It has been shown that exogenous Tβ4 plays an anti-apoptotic role in ethanol-stimulated corneal epithelial cells by inhibiting the release of cytochrome c from the mitochondria and suppressing the activation of caspases [48]. To study the effect of Tβ4 on apoptosis in the current infectious model, we assessed total cytoplasmic cell death and multiple critical enzymes involved in the apoptotic pathway (Figure 7A–E). Histone-associated DNA fragments generated subsequent to cell death were detected at 3 days p.i. (A). The results suggest that both the Tβ4 alone and adjunctive Tβ4 treatment groups significantly inhibited cell death when compared to the PBS control. Surprisingly, ciprofloxacin treatment exhibited the most apparent reduction in cell death when compared to all other groups. Levels of caspase-8, an extrinsic apoptotic pathway enzyme, were measured to explore the activation of initiator caspases during apoptosis (B). We found that Tβ4 only treatment significantly boosted the production of caspase-8 when compared to other treatment groups. However, both ciprofloxacin and adjunctive Tβ4 treatments significantly downregulated the expression of caspase-8. To elucidate the converging roles of downstream effector caspases in inciting apoptotic cell death, caspase-3 activity (C) was assessed, as well. Levels of caspase-3 were significantly inhibited in both Tβ4 only and adjunctive Tβ4 treatment groups when compared to both the PBS controls and ciprofloxacin treatment. In addition, Bax (D), a proapoptotic molecule involved in the intrinsic pathway, was uniformly expressed among the PBS control, Tβ4 only, and ciprofloxacin treatment groups. However, it was significantly decreased with adjunctive Tβ4 treatment when compared to all other groups. Bcl-2 (E), an antiapoptotic molecule in the intrinsic apoptosis pathway, was similarly expressed in the PBS control, Tβ4 only, and adjunctive Tβ4 treatment groups but significantly upregulated with the ciprofloxacin only treatment. These findings indicate that adjunctive Tβ4 treatment represses apoptosis by regulating aspects of both the extrinsic and intrinsic apoptotic pathways. 

### 3.8. Adjunctive Tβ4 Treatment Regulates Apoptosis In Vitro

**Figure 8:**
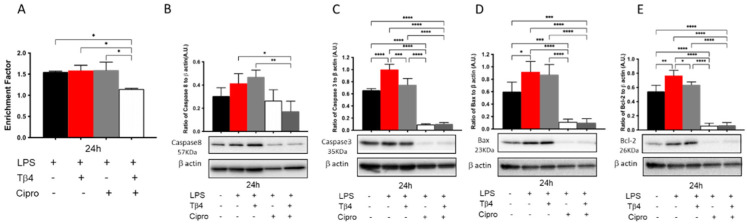
In vitro assessment of apoptotic activity following LPS-induced stimulation of peritoneal-derived PMNs. Cytoplasmic DNA fragments (**A**), caspase-8 (**B**), caspase-3 (**C**), Bax (**D**), and Bcl-2 (**E**) were assessed after Tβ4, cipro, and adjunct Tβ4 treatments. Levels as detected by Western blot are presented as a ratio to β-actin ± SD. *n* = 3; * *p* < 0.05, ** *p* < 0.01; *** *p* < 0.001, and **** *p* < 0.0001.

The effect of Tβ4 on apoptosis was further confirmed in vitro using LPS-stimulated peritoneal PMNs from B6 mice at 24 h. As shown in Figure 8, LPS stimulation did not increase cell death (A) or caspase-8 (B); however, levels of caspase-3 (C), Bax (D), and Bcl-2 (E) were significantly increased following LPS stimulation compared to media only controls. The adjunctive Tβ4 treatment of LPS-stimulated PMNs displayed significantly decreased cell death when compared to all treatment groups (A). Regarding caspase-8 (B), adjunctive Tβ4 treatment significantly decreased LPS-induced levels. Caspase-3 (C), Bax (D), and Bcl-2 (E) were all significantly decreased following ciprofloxacin only and adjunctive Tβ4 treatments, while Tβ4 only treatment significantly downregulated the activation of caspase 3 (C) and Bcl-2 (E) following LPS stimulation. Combined with the in vivo results, these findings further reveal that adjunctive Tβ4 treatment effectively influences PMN apoptosis. 

## 4. Discussion

*P. aeruginosa*-induced keratitis progresses rapidly causing corneal opacity that threatens vision acuity. Despite that traditional ophthalmic fluoroquinolones target the invading pathogen, host tissue inflammation remains unmanageable leading to immunopathological damage [49]. Sosne et al. have established that Tβ4 exerts an anti-inflammatory role in ocular surface diseases [24,25,50,51]. Our lab has extended upon this work revealing an immunoregulatory role that improves the disease response during microbial keratitis [30]. In this regard, we have recently published evidence that Tβ4 exerts this immunoregulatory effect, partially by modulating MΦ infiltration, activation, and function in the *P. aeruginosa*-infected mouse model [33], which is the other cell population (CD45^+^Ly6G^−^F4/80^+^) that was detected by our flow cytometric analysis. Understanding the effect Tβ4 exerts upon on MΦ cellular function is important; however, PMNs are the major cellular infiltrate into the cornea following infection. Therefore, studying PMN effector cellular function is an integral part to fully understanding the therapeutic effects of Tβ4 regarding resolution of corneal inflammation and infection. Though PMNs have been studied in the context of microbial keratitis, the results from the current study reveal the novel findings that that adjunctive Tβ4 treatment immunoregulates the host response by decreasing the infiltration and activation state of PMNs, while enhancing anti-inflammatory markers, inhibiting ROS generation, downregulating NETosis, and regulating PMN apoptosis. Further, these findings suggest that Tβ4 does not exert a generalized effect on inflammatory infiltrates, but instead regulate these two cell types (PMN and MΦ) on a more discriminatory level.

Overall, adjunctive Tβ4 treatment reduced PMN infiltration into the infected cornea with a phenotypic shift from pro-inflammatory toward anti-inflammatory. Of note is the significant reduction in all pro-inflammatory markers—CD13, CD177, CD80, and CD63. CD177, which is thought to be expressed exclusively on PMNs and upregulated during infection, has also been demonstrated to mediate PMN-endothelial cell interaction to promote PMN transmigration by binding platelet endothelial cell adhesion molecule-1 [52]. CD63 is an essential cofactor to P-selectin, and they act together in recruiting leukocytes. A lack of CD63 results in a failure of leukocyte extravasation and recruitment [53]. The significantly decreased expression of CD177 and CD63 in the adjunctive Tβ4 treatment group directly contributes to reduced PMN infiltration, augmenting improved disease outcome. It has been reported that prolonged wound stress-induced toxicity can significantly increase the expression of CD80 on PMNs [54]. This report correlates with our finding that CD80 was highly expressed in the PBS control and Tβ4 only treatment groups, both of which displayed severe and sustained inflammation. CD13 plays a role in the degradation of soluble peptide mediators and increases during PMN maturation and apoptosis [55]. It was significantly reduced in the infected cornea following adjunctive Tβ4 treatment, supporting the reduction in PMN infiltration while also suggesting that Tβ4 may influence PMN apoptosis. In fact, this is consistent with previous clinical observations [30] and further demonstrated by the functional work carried out in the current study. Regarding anti-inflammatory molecules, AnxA1 is an endogenous glucocorticoid-regulated protein that counter-regulates inflammatory events to restore homeostasis [56]. Monocytes and PMNs highly express CD192, which functions together with CXCR1, CCR1, and CCR5 to enhance PMN mobilization and degranulation [57]. CD206 has been reported as an anti-inflammatory cell surface marker for both MΦ and PMNs with a role in resolving inflammation and wound repair [58,59]. The upregulation of AnxA1, CD192, and CD206 in the Tβ4 treatment groups stimulates the resolution of inflammation and host immune homeostasis. These observed changes in activation state may also be associated with the maturation state of neutrophils. Though it is known that neutrophil precursors within the bone marrow mature into circulating blood neutrophils that typically become activated upon tissue extravasation, it is possible (and expected) that those PMNs migrating into the corneas of Tβ4 + ciprofloxacin-treated mice are a subset of cells that carry out other functions beyond degranulation and apoptosis. In this regard, reverse migration or senescence would reflect some of these additional, potential functions. 

Overwhelmed PMN infiltration and upregulated expression of pro-inflammatory molecules contribute to the typically susceptible disease response observed in B6 mice following infection. However, large amounts of ROS and subsequently induced lipid damage play critical roles in the pathogenesis of microbial keratitis, as well. ROS are produced by the respiratory oxygen chain in physiological conditions and activate cellular signaling for survival [43]. Persistent recruitment of PMNs results in ROS overproduction has been recognized as an essential factor in the pathogenesis of ocular surface diseases [60]. Somewhat unexpected, Tβ4-treated corneas and isolated PMNs exhibited significant upregulation of ROS production. However, the adjunctive Tβ4 treatment inhibited the generation of ROS both in vivo and in vitro. It is worth noting that superoxide, an important component of ROS, was reduced by the Tβ4 only treatment in the in vivo model, yet further reduced by the adjunctive Tβ4 treatment in both in vivo and in vitro models. Overproduction of ROS results in oxidative damage to the cells, especially lipid peroxidation of the cell membrane. HEL adducts are produced during the early stages of lipid peroxidation [46]. We revealed that Tβ4 only treated corneas and isolated PMNs resulted in similar levels of lipid peroxidation damage when compared to the positive controls. However, ciprofloxacin and adjunctive Tβ4 treatments tremendously inhibited ROS-induced lipid peroxidation in the infected B6 mouse corneas and LPS-stimulated PMNs. Adjunctive treatment exhibited a robust function in inhibiting the overproduction of ROS and induced lipid damage, which lower levels are beneficial for the inflamed tissues to maintain a balanced immune response. Combined with our previous finding that Tβ4 regulates reactive nitrogen species to improve immune response [33], the current study further demonstrates that Tβ4 influences ROS signaling to modulate inflammation and limit oxidative damage. This is a key feature in the development of a therapeutic to treat inflammatory diseases in that ROS needs to be reduced, but maintained at homeostatic levels.

NETs are web-like DNA structures decorated with histones and cytotoxic proteins released by activated PMNs to trap and neutralize pathogens during the innate immune response. However, NETosis can lead to detrimental effects on the host, as well, as observed in several autoimmune diseases that are associated with high rates of NETosis and/or defects in NET clearance [61]. Thus, understanding NETosis in the current paradigm not only provides mechanistic insight regarding how Tβ4 functions, but also contributes to our understanding as to how this process contributes to the disease response. Both in vivo and in vitro results indicate that adjunctive Tβ4 treatment efficiently inhibits NET formation, which is beneficial to limit damage to the cornea. The contribution of NE has been evaluated in mouse models of sterile inflammation and demonstrated to play a role in NETosis triggered by microbes, but is not required for PMN NET production in vitro with noninfectious stimuli [62]. We evaluated the correlation of NE and NETosis in *P. aeruginosa*-infected corneas and found a positive relation between NE expression and NETosis activity. However, when the protein levels of NE were measured following LPS stimulation of PMNs in vitro, there was no correlation. NE may not be required for PMNs to form NETs in vitro following LPS stimulation. Or perhaps NE levels are induced by a different component of the bacteria other than LPS. To this end, additional studies need to be conducted to test these possibilities. Conversely, significant downregulation of NETosis activity in ciprofloxacin and adjunctive Tβ4 treatments was consistent with the observed mild clinical outcome in these two groups.

It has been demonstrated that exogenous Tβ4 treatment inhibits apoptosis and is favored in several circumstances, including kidney fibrosis [63], liver cirrhosis [64], oxidative damage to myocardium [65], and intervertebral annulus cells [66]. When human corneal epithelial cells are challenged with external stress, Tβ4 represses the release of cytochrome c from the mitochondria and suppresses the subsequent activation of caspases [48]. Significantly decreased cytoplasmic histone-associated DNA fragments revealed that Tβ4 inhibits cell death in adjunctive Tβ4-treated corneas and isolated PMNs. Furthermore, we found that Tβ4 exhibits a robust antiapoptotic function in the PMNs by inhibiting proapoptotic enzymes caspase-3, caspase-8, and pro-apoptotic protein Bax, as well as the anti-apoptotic protein, Bcl-2. Considering that apoptosis could be a protective phenomenon in the pathogenesis of inflammation, to some extent it was somewhat unexpected that apoptosis was reduced in the adjunctive Tβ4 treatment groups. Apoptosis of PMNs following successful phagocytosis (of bacteria, in this case) is an essential part of the resolution of inflammation though. The balance between apoptosis and cell survival, the tissue milieu, and the timing of apoptosis are critical in an immune defense [18]. Zhou et al. provide evidence that the delayed apoptosis of PMNs in the cornea after *P. aeruginosa* infection leads to exacerbated disease outcomes [67]. We have also shown that PMNs are not undergoing apoptosis, but necrosis in the infected corneas of B6 mice, which contributes to the sustained chronic state of inflammation observed in these animals [39]. Therefore, we expect that apoptotic activity with the adjunctive Tβ4 treatment is enhanced at earlier time points of the infection (e.g., one or two days p.i.) and then becomes diminished as inflammation wanes, and resolution is underway by day three p.i. In contrast, apoptotic activity was still intense in the PBS control and Tβ4 only groups, likely due to the persistent state of inflammation and dysregulated immune response. It also suggests that necrotic cell death and apoptotic cell death could happen simultaneously in the infected cornea [68], whereby, the former contributes to destructive necrosis in the *P. aeruginosa* infected corneas. Clinical observations and histology results show that corneas receiving the adjunctive Tβ4 treatment were almost completely healed from infection [30]. In addition, apoptotic cells are rarely detected under physiological conditions. These together might explain the low expression of apoptosis enzymes and anti-apoptotic enzymes in the adjunctive Tβ4 treated group, as these corneas are already resuming corneal homeostasis. 

Our previous work investigating adjunctive Tβ4 treatment in *P. aeruginosa*-infected corneas has revealed that adjunct Tβ4 treatment affects lipoxygenase enzyme expression, RNS generation, and efferocytosis activity [32,33]. The current study continued this examination by focusing on the PMNs and related functions. The well-regulated inflammatory response observed following the adjunct Tβ4 treatment stems from not only Tβ4-induced effects but also the synergism with ciprofloxacin. This synergistic effect was not observed to the same extent regarding MΦ activation and function, suggesting that Tβ4 was influencing these two inflammatory cell types differently. Provocative leads include whether PMNs express the P2X7 receptor, which is known to bind Tβ4; if Tβ4 is acting as a transcription factor as previously indicated, or if the full effects of Tβ4 require cell–cell interactions that include the PMNs, MΦ and/or corneal epithelial cells. 

We have previously described the ciprofloxacin-induced effects in the context of MΦ activation and effector function following corneal infection [33]. Yet given the widespread use of antibiotics and the observed off-target effects regarding PMN function as well, it is worth reiterating herein. Ciprofloxacin is a widely used fluoroquinolone to treat bacterial infections by inhibiting bacterial DNA gyrase and DNA topoisomerase [69]. While some side effects have been noted (e.g., sensitivity to sun and gastrointestinal issues), the influence of antibiotics on the immune system is not well understood. The current work expands our previous finding that ciprofloxacin enhances the inflammatory state of not only MΦ but also PMNs—two major cell types involved in the innate immune response. Herein, we demonstrate a shift in the PMN activation state toward inflammation (increased pro-inflammatory markers/decreased anti-inflammatory markers) following ciprofloxacin treatment versus adjunct Tβ4 or Tβ4 alone treatments. In addition, our in vitro studies revealed that ciprofloxacin was found to be toxic to MΦ, PMNs, and human corneal epithelial cells at the standard working concentration of 0.3%, which was then reduced to 0.03%. These effects suggest a mechanism that goes beyond the inhibition of DNA replication and warrant further investigation. On the other hand, ciprofloxacin demonstrated a protective effect regarding ROS generation and lipid peroxidation that did not appear to be synergistic with Tβ4. The development of an adjunct therapy, such as Tβ4, that could allow for the use of antibiotics at lower concentrations would not only help to reduce antibiotic resistance, but also unwanted side effects on the host immune response, while preserving protective effects. 

In summary, we demonstrated a novel regulatory role for Tβ4 regarding PMN cellular function as indicated in the in vivo *P. aeruginosa*-induced mouse model of keratitis and in vitro mouse-derived PMNs and as evidenced by the regulation of ROS production, NETosis, and apoptosis. Adjunctive Tβ4 treatment influences PMNs during the inflammatory response to resolve the host immune response and improve disease outcome, which substantially reinforces its clinical applicability for the treatment of bacterial keratitis. Most exciting, these studies will serve as the basis for Tβ4 in future clinical trials of infectious keratitis. 

## Data Availability

The data presented in this study are available in the current article and within the Supplementary Materials.

## Supplementary Materials

The following are available online at https://www.mdpi.com/article/10.3390/cells10123579/s1, Figure S1: Gating Strategy Used to Identify PMN.
