# Supplementary material for: Adjunctive Thymosin Beta-4 Treatment Influences PMN Effector Cell Function during Pseudomonas aeruginosa-Induced Corneal Infection
Source: Cells. 2021 Dec 18;10(12):3579. doi: 10.3390/cells10123579 (PMC8700181; doi:10.3390/cells10123579)
Supplement: Supplementary file 1 [file cells-10-03579-s001.zip › cells-1481103-supplementary.pdf]

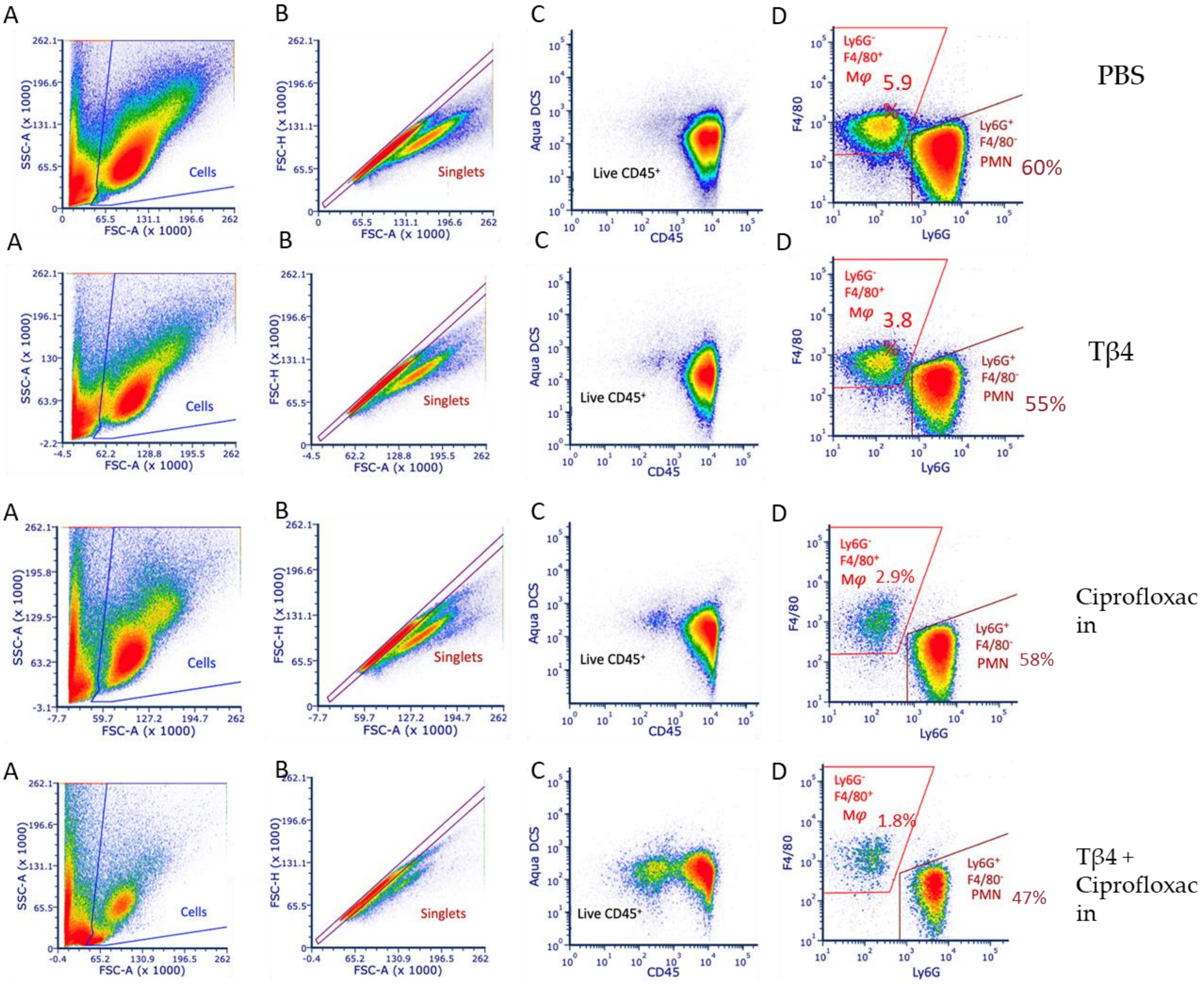

Figure S1. Gating strategy used to identify PMN. **Panel A**: Forward and side scatter density plots identify cell population of interest and excluding debris;. **Panel B**: from gating A, Forward scatter height(FSC-H) versus forward scatter area density (FSC-A) plot further characterized the cell populations to exclude doublet;. **Panel C**: From gating B, the singlets are characterized by CD45 and LIVE/DEADTM Fixable Aqua dead cell stain to get live CD45+ cell population. **Panel D**: Live CD45+ cells from gating C are further labeled by Ly6G and F4/80 to discriminate PMN from M*φ*.
